# A therapeutic vaccine strategy to prevent *Pneumocystis* pneumonia in an immunocompromised host in a non-human primate model of HIV and *Pneumocystis* co-infection

**DOI:** 10.3389/fimmu.2022.1036658

**Published:** 2022-12-06

**Authors:** Whitney Rabacal, Finja Schweitzer, Heather M. Kling, Lizabeth Buzzelli, Emily Rayens, Karen A. Norris

**Affiliations:** ^1^ Center for Vaccines and Immunology, Department of Infectious Diseases, University of Georgia, Athens, GA, United States; ^2^ Department of Immunology, University of Pittsburgh, Pittsburgh, PA, United States

**Keywords:** *Pneumocystis*, *Pneumocystis* pneumonia (PCP/PJP), fungal vaccine, α-galactosylceramide (α-GC, α-GalCer, KRN7000), HIV/SIV, immunocompromised, and non-human primate (NHP)

## Abstract

**Introduction:**

*Pneumocystis* is a ubiquitous fungal pathogen that causes pneumonia (PCP) and pulmonary sequelae in HIV-infected individuals and other immunocompromised populations. With the success of anti-retroviral therapy for HIV-infected individuals the frequency of PCP in that population has decreased, however, PCP remains a significant cause of morbidity and mortality in individuals with hematologic and solid malignancies, and in individuals treated with immunosuppressive therapies for autoimmune diseases, and following bone marrow and solid organ transplantation. Despite the clinical need, there is no approved vaccine to prevent PCP in vulnerable populations. The ultimate goal of the field is to develop an effective vaccine that can overcome immune deficits in at risk populations and induce long-lasting protective immunity to *Pneumocystis*. Toward this goal, our laboratory has established a model of PCP co-infection in simian immunodeficiency virus (SIV)-infected non-human primates (NHP) and identified a recombinant protein sub-unit vaccine, KEX1, that induces robust anti-*Pneumocystis* immunity in immune-competent macaques that is durable and prevents PCP following simian immunodeficiency virus (SIV)-induced immunosuppression. Type I, or invariant natural killer T (iNKT) cells have the potential to provide B cell help under conditions of reduced CD4+ T cell help.

**Methods:**

In the present study, we used the SIV model of HIV infection to address whether therapeutic vaccination with the iNKT cell-activating adjuvant α-galactosylceramide (α-GC) and KEX1 (α-GC+KEX1) can effectively boost anti-*Pneumocystis* humoral immunity following virus-induced immunosuppression.

**Results:**

Immunization of antigen-experienced NHPs with α-GC+KEX1 during the early chronic phase of SIV-infection significantly boosted anti-*Pneumocystis* humoral immunity by increasing memory B cells and antibody titers, and enhanced titer durability during SIV-induced immunosuppression. This therapeutic vaccination strategy boosted anti-*Pneumocystis* immune responses during SIV-infection and contributed to protection against *Pneumocystis* co-infection in KEX1-vaccinated macaques.

**Conclusion:**

These studies present a novel strategy for stimulating durable anti-*Pneumocystis* humoral immunity in the context of complex, chronic SIV-induced immunosuppression and may be further applied to immunization of other immunosuppressed populations, and toward other common recall antigens.

## Introduction

*Pneumocystis* is an opportunistic fungal pathogen and the causative agent of life-threatening *Pneumocystis* pneumonia (PCP) in HIV-infected and other immunocompromised populations. *Pneumocystis* colonization has been associated with a number of chronic lung diseases including chronic obstructive pulmonary disease (1), severe asthma (2, 3), and cystic fibrosis (4). Despite decreases in the incidence of PCP in HIV-infected individuals with access to anti-retroviral therapy and *Pneumocystis* prophylaxis, PCP continues to be a serious AIDS-defining opportunistic infection. HIV-infected populations who have remained at risk of PCP in the post-anti-retroviral therapy (ART) era include individuals who are undiagnosed, do not have access to ART, are unable to tolerate ART, in whom ART is not effective, and those with CD4+ T cell counts less than 200 cells/ul (5). Within these populations, PCP continues to be a leading cause of morbidity and mortality, especially in resource limited settings. Currently, populations with the highest incidence of PCP are those receiving immunosuppressive therapies for treatment following organ transplantation, solid and hematopoietic malignancies, and autoimmune diseases. Even when anti-*Pneumocystis* treatment is initiated, mortality remains high among HIV-infected persons (10-40%) and non-HIV infected populations (30-60%) (6). Trimethoprim sulfamethoxazole (TMP-SMX) is an effective prophylactic and therapeutic against *Pneumocystis*, but this treatment strategy remains problematic due to drug interactions, treatment-limiting adverse events, break-through PCP despite prophylaxis, and increased concerns of emerging drug resistance (7–9). In addition, treatment cannot reverse permanent obstructive lung damage associated with PCP recovery (10, 11). Due to the high rate of mortality with the current standard of care, PCP remains a significant infection among immunosuppressed populations.

*Pneumocystis* natural airborne exposure, asymptomatic colonization, and clearance are common in immunocompetent populations. Through repeated environmental exposure, most individuals develop positive serology against *Pneumocystis* antigens during childhood (12–15). Our laboratory and others have investigated the immune responses to *Pneumocystis* in healthy individuals, immunocompromised patients including HIV-infected individuals, persons with pulmonary disease (chronic obstructive pulmonary disease (COPD), severe asthma, tobacco smokers, and in non-human primate (NHP) models of PCP (1–3, 10, 11, 16–18). Several studies have demonstrated the importance of antibodies against the *Pneumocystis* protein antigen KEX1 in promoting protection against PCP and *Pneumocystis*-related pulmonary sequalae (1, 17, 19, 20). In both HIV-infected individuals and simian immunodeficiency virus (SIV)-infected NHPs, high antibody titers against KEX1 but not a distinct *Pneumocystis* surface glycoprotein, MSG, correlated with a reduced incidence of PCP (17, 20). In studies of other chronic pulmonary diseases, persistent *Pneumocystis* colonization has been associated with the development of COPD (1, 16, 18) and severe asthma (3). Low or undetectable KEX1 antibody titers in individuals with COPD and severe asthma are associated with increased disease severity (1, 2), further emphasizing the significance of KEX1 specific antibodies in anti-*Pneumocystis* surveillance. Through the use of a NHP model of HIV and *Pneumocystis* co-infection, Kling et al. demonstrated that vaccination with a recombinant protein subunit of KEX1 and alum induces robust boosting of anti-*Pneumocystis* humoral memory in immune competent macaques that is protective against PCP following chronic SIV-induced immunosuppression (19). However, in both HIV-infected and non-HIV infected PCP-susceptible populations, deficiencies in CD4+ T cell populations and immunologic dysfunction can limit humoral responses toward common recall antigens. Therefore, novel vaccination strategies in concert with novel antigen candidates are necessary to restore long-lasting protective immunity against *Pneumocystis* in at-risk populations.

Type I invariant natural killer T (iNKT) cells are innate-like lymphocytes that recognize glycolipid antigens presented by the major histocompatibility-like molecule CD1d expressed on antigen presenting cells (APC) (21, 22). Upon recognition of glycolipid cognate antigens, activated iNKT cells secrete a wide array of cytokines that can drive both innate and adaptive immune responses (21, 23, 24). The marine sponge glycosphingolipid α-galactosylceramide (α-GC) is a potent agonist of iNKT cell antigen that has been increasingly studied for its use as an adjuvant. In the context of vaccination, α-GC activated iNKT cells have been reported to promote B cell proliferation and antibody secretion (25–28); however, the ability of iNKT cells to promote long-lived B cell memory responses remains controversial (29). In murine studies, vaccination of MHC class II deficient animals with α-GC against protein antigens have been demonstrated to elicit IgG responses, suggesting that in the absence of CD4+ T cell help, iNKT cells can provide direct B cell help, regulate the formation of memory, and promote antigen recall responses (26, 30). These data represent a promising approach to induce both the formation of humoral memory and potent antibody recall responses in immunosuppressed populations with severely reduced CD4+ T cell help. Although the adjuvant properties of α-GC in immunocompetent systems have been documented, its use during uncontrolled HIV-infection to promote humoral boosting against a recall antigen has yet to be explored. Herein, we used the SIV model of HIV infection to test the capacity of a therapeutic vaccination strategy to promote humoral recall responses against the *Pneumocystis* antigen KEX1, and protective immunity in rhesus macaques with established memory, under conditions of SIV-immunosuppression and uncontrolled viremia.

## Materials and methods

### Animals

Adult rhesus macaques (*Macaca mulatta*) of Indian (Cohort 1, n=16) and Chinese (Cohort 2, n=14) origin aged 5-9 years old were obtained from vendors approved by the University of Georgia Department of Animal Resources and housed in accordance with the *NIH Guide for the Care and Use of Laboratory Animals* (31) in ABSL2+ primate facilities. Prior to admission to the study, all animals were screened and found negative for simian retroviruses (SIV, SRV, and STLV). All procedures were approved by the Institutional Animal Care and Use Committee at the University of Georgia.

### Immunization of macaques and SIV challenge

A 270-nucleotide fragment of macaque-derived *Pneumocystis* KEX1 (accession no. EU918304) was cloned into the pET-28b(+) expression vector (Novagen) and recombinantly expressed in Escherichia coli BL21(DE3) *pLys* (ThermoFisher) containing the plasmid; recombinant protein was purified by affinity chromatography as previously described (19, 32). Recombinant KEX1 was used for immunization, enzyme-linked immunosorbent assay (ELISA), and enzyme-linked immunospot (ELISpot) assay. Healthy rhesus macaques were randomly assigned into one of two groups for immunization with the 11kDa subunit KEX1 recombinant protein subunit vaccine. Eight rhesus macaques of Indian origin and six macaques of Chinese origin were intramuscularly immunized with 100µg of KEX1 and alum (Imject Alum, Thermo Scientific) (alum+KEX1) mixed in a 1:1 ratio at 16 and 8 weeks prior to SIV infection. Eight rhesus macaques of Indian origin and eight macaques of Chinese origin were sham vaccinated with alum and PBS (alum+PBS) at corresponding timepoints. All cohorts were infected with SIV/Delta B670 (1:100 in PBS) (33, 34), tissue culture infectious dose of 50% (TCID_50_) = 2.6 x10^5^), intravenously (35, 36). Following 10 weeks of SIV-mediated CD4+ T cell depletion, animals were then therapeutically boosted with 100µg of KEX1 and 5µg α-GC (Cayman Chemical) (α-GC+KEX1) mixed in up to 500µl PBS (Cohorts 1 and 2 VAX) or sham vaccinated with α-GC and PBS (α-GC+PBS) (Cohort 1 Sham) or PBS alone (Cohort 2 Sham) at 10 and 14 weeks post-infection. An α-GC stock solution was prepared by dissolving the powder in 1mg/ml DMSO and stored at -80°C prior to vaccine preparation. Animals that were incompatible with evaluating the efficacy of α-GC+KEX1 boosting in the context of SIV-induced immunosuppression, such as those that exhibited rapid clinical decline due to SIV or unrelated disease, or were classified as long-term non-progressors, were removed from the study (**Extended Data Tables 1**, **2**). Among the remaining animals, our analysis included 12 macaques of Indian origin (Cohort 1; VAX n=7; Sham n=5) and 13 macaques of Chinese origin (Cohort 2; VAX n=6; Sham n=7). Blood and bronchoalveolar lavage (BAL) fluid samples were collected at baseline and following vaccination. Plasma and BAL supernatants were collected and stored at -80°C until evaluation. Plasma viral titers and peripheral blood CD4+ T cells were monitored weekly within the first 4 weeks of infection and monthly thereafter up to 36 weeks post-infection to assess progression of SIV infection, as previously described (19).

### ELISA and ELISPOT assays

To monitor anti-KEX1 IgG antibody levels in response to vaccination, plasma samples were evaluated by ELISA as previously described (19, 32). To monitor peripheral blood samples for the presence of KEX1-specific memory B cells (KEX1+ IgG memory B cells), memory B cell ELISPOT assays were performed using mitogen stimulated fresh peripheral blood lymphocytes as previously described (19).

### Flow cytometry

Peripheral blood was processed for flow cytometry as previously described (17, 37). Anti-CD3 (SP34), anti-CD4 (L200), anti-CD8 (RPA-T8), anti-CD14 (M5E2), anti-CD80 (L307.4), anti-CCR4 (1G1), anti-HLADR (G46-6), and anti-Vα24-Jα18 (6B11) antibodies were purchased from BD Biosciences. Anti-CD11b (ICRF44), anti-CD11c (3.9), anti-CD20 (2H7), anti-CD86 (FUN-1), anti-CXCR3 (G025H7), and anti-Vα24 (C15) antibodies were purchased from Biolegend (San Diego, CA). Human CD1d PBS-57 tetramer and human CD1d unloaded control tetramer were obtained from the NIH Tetramer Core Facility (Atlanta, GA). Dendritic cells were identified as CD11b+CD11c+HLADR+CD14- in Cohort 1 and CD11b+CD11c+HLADR+CD14-CD20- in Cohort 2. iNKT cells were identified as 6B11(Vα24-Jα18)+CD3+ in Cohort 1 and Vα24+TET(CD1d PBS-57 tetramer)+CD3+ in Cohort 2. Tetramer specific staining was verified against samples stained with unloaded CD1d control tetramers. CD4+ T helper type 1 (Th1) and T helper type 2 (Th2) subsets were identified as CD4+CD8-CXCR3+CCR4- and CD4+CD8-CXCR3-CCR4+ as previously described (19, 38). Standard flow cytometric procedures were used to acquire data on a LSRII flow cytometer (BD Biosciences). Analysis was performed using FlowJo analysis software (Tree Star, Inc., Ashland, OR).

### *Pneumocystis* challenge and evaluation of *Pneumocystis* co-infection

*Pneumocystis* cannot be reliably cultured *in vitro*. *Pneumocystis* co-infection of SIV-infected macaques was performed *via* natural airborne transmission by cohousing with *Pneumocystis* infected animals, as described (17, 19, 39). Following SIV infection, *Pneumocystis* co-infection status was evaluated monthly by the detection of the *Pneumocystis* mitochondrial large subunit rRNA gene by PCR (first-round PCR) in BAL cell lysate (32, 37). To control for DNA quality in BAL fluid samples, a PCR for β-globin was used as a control (39). A diagnosis of *Pneumocystis* co-infection was made through the detection of *Pneumocystis* DNA in BAL fluid or terminal lung homogenate by first-round PCR, or by immunohistochemistry staining of formalin fixed paraffin embedded lung sections with anti-*Pneumocystis* clone 3F6 (40), as previously described (19).

### Statistical analysis

All statistical analyses were performed using GraphPad Prism (GraphPad Software, La Jolla, CA). To assess significant differences in KEX1-specific titers and KEX1 memory B cells between indicated timepoints following vaccination within KEX1-immunized groups, Wilcoxon signed rank tests were performed. Serial group characterizations of CD4+ T cell frequency, viral load, iNKT cell numbers, dendritic cell activation, and T helper skewing, were analyzed using repeated measures mixed modeling using the Geisser–Greenhouse model correction and Sidak correction for multiple comparisons. Mantel-Cox test was used to analyze *Pneumocystis* co-infection incidence curves.

## Results

### Humoral immune responses in healthy rhesus macaques and following therapeutic boosting during the early chronic phase of SIV infection

In this study, we hypothesized that anti-*Pneumocystis* humoral immunity could be therapeutically boosted in SIV-immunosuppressed animals with a novel vaccine strategy using α-GC+KEX1. As an initial proof of concept study, we sought to evaluate the efficacy of α-GC+KEX1 vaccination in animals with established KEX1-specific memory during the early chronic phase of SIV-infection. Prior to immunization, plasma samples from cohorts of healthy rhesus macaques were evaluated for evidence of prior *Pneumocystis* exposure. We have previously established that antibody titers that were above 10^4^ KEX1 IgG RET in healthy macaques correlate with protection against natural *Pneumocystis* colonization following SIV-infection (17). At baseline, all NHPs in both cohorts had mean plasma RETs below the *Pneumocystis* correlate of protection at 10^4^ KEX1 IgG RET, reflecting the presence of low, pre-existing KEX1-specific humoral immunity and susceptibility following SIV-infection if left unvaccinated.

**Figure 1:**
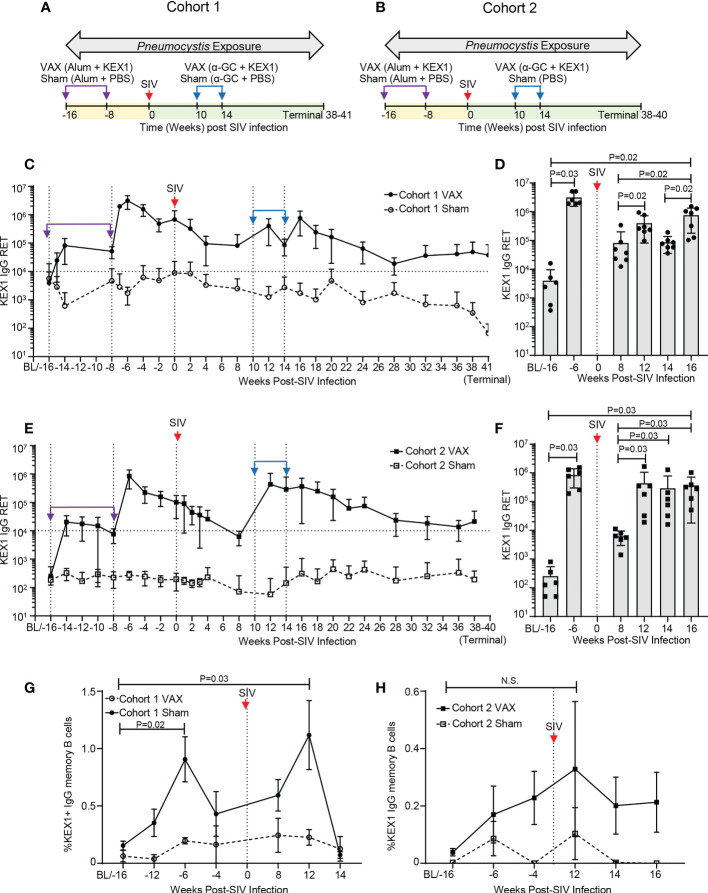
KEX1-specific humoral responses in rhesus macaques following vaccination with alum+KEX1 prior to SIV infection and boosting with α-GC+KEX1 in the early chronic phase of infection. Schematic of immunization, SIV infection, and *Pneumocystis* co-infection of rhesus macaques in **(A)** Cohort 1 and **(B)** Cohort 2. Vertical dashed lines indicate vaccination timepoints. Purple arrows and indicate immunizations with in healthy macaques with alum+KEX1 prior to SIV infection. Blue arrows indicate therapeutic boosting with α-GC+KEX1 at 10 and 14 weeks post-infection. Kinetics of plasma KEX1-specific reciprocal endpoint titers (KEX1+ IgG RET) in **(C, D)** Cohort 1 and **(E, F)** Cohort 2. Horizontal dashed lines at 10,000 RET indicate the correlate of protection. **(D, F)** Peak plasma titers following alum+KEX1 immunization and α-GC+KEX1 boosting. **(G, H)** Kinetics of KEX1-specific memory B cell responses (%KEX1 IgG Memory B cells) Not significant (N.S.). **(D, F, G, H)** Wilcoxon signed rank test were performed to compare indicated timepoints within the VAX group. Data represents the mean ± SD.

To ensure established KEX1-specific B cell memory in experimental cohorts, healthy rhesus macaques were vaccinated with alum+KEX1 16 and 8 weeks prior to SIV-infection. NHPs were then infected with SIV and therapeutically boosted with α-GC+KEX1 at 10 and 14 weeks post-infection (**Figure 1A**; Cohort 1). This experiment was then repeated in a second cohort (**Figure 1B**; Cohort 2). There were no significant differences in age or weight at the time of SIV infection between each of the VAX and Sham treated groups within each cohort analyzed (**Extended Data Tables 1**, **2**). In healthy KEX1-immunized animals, plasma KEX1 IgG titers peaked 2 weeks after the primary vaccination series in both cohorts (-6 weeks post-infection; Cohort 1 VAX 3,035,429 ± 1,449,501; Cohort 2 VAX 824,667 ± 543,686; **Figures 1C–F**; **Extended Data Tables 1**, **2**), mirroring our previous studies of vaccination with alum+KEX1 in healthy macaques (19, 32). Following SIV infection, KEX1 titers declined with a nadir observed at 8 weeks post-infection in both Cohort 1 (VAX; 81,688 ± 117,197; **Figures 1C, D**; **Extended Data Table 1**) and in Cohort 2 (VAX; 6,233 ± 3,275; **Figures 1E, F**) where, the titer nadir notably dropped slightly below the correlate of protection (17).

We then tested whether therapeutic boosting with α-GC+KEX1 during the early chronic phase of SIV-infection at 10 and 14 weeks post-infection could boost KEX1-specific humoral immunity. Within the vaccinated group of Cohort 1 (Cohort 1 VAX), a robust peak was observed at 16 weeks post-infection (754,819 ± 575,821; **Extended Data Table 1**) following both doses of α-GC+KEX1, boosting titers approximately 9.2-fold between 8 and 16 weeks post-infection (**Figure 1D**, P=0.02). Mean titers were maintained above the correlate of protection throughout chronic SIV infection until study termination (37,440 ± 50,991; **Figure 1C**). In comparison, mean plasma titers in the sham vaccinated group (Cohort 1 Sham) remained below the correlate of protection from baseline until study termination. Peripheral blood KEX1-specific memory B cell responses (KEX1 IgG memory B cells) displayed a peak 12 weeks post-infection following the first therapeutic dose of α-GC+KEX1 (**Figure 1G**); however, we were unable to evaluate memory B cell responses following the second vaccination at 16 weeks post-infection due to insufficient cell numbers. In contrast to Cohort 1 VAX, peak titers in the vaccinated group of Cohort 2 (Cohort 2 VAX) were observed at 12 weeks post-infection (433,067 ± 620,472; **Extended Data Table 2**), following a single dose of α-GC+KEX1. Despite the more pronounced decline in titers within the Cohort 2 VAX group observed at 8 weeks post-infection nadir, the first therapeutic dose of α-GC+KEX1 boosted titers approximately 69.5-fold between 8 and 12 weeks post-infection (**Figure 1F**, P=0.03). Mean KEX1 titers remained above the correlate of protection until study termination (21,200 ± 28,124; **Figure 1E**). Similarly, KEX1 IgG memory B cells peaked in Cohort 2 VAX at 12 weeks post-infection (**Figures 1G, H**), and mirrored the robust peak in KEX1 IgG titers observed at the same timepoint. Together, these data demonstrate that therapeutic vaccination with α-GC+KEX1 induces robust boosting of KEX1-specific antibody titers and memory B cells that are maintained throughout the course of untreated SIV infection.

### Effects of therapeutic boosting with α-GC+KEX1 on peripheral blood CD4+ T cells and SIV infection

**Figure 2:**
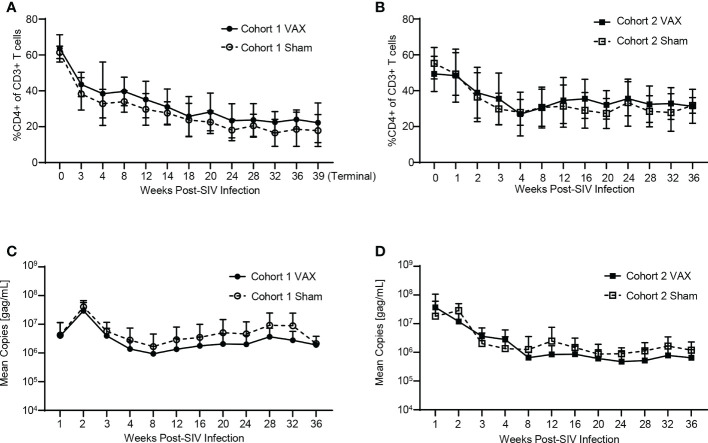
Evaluation of peripheral blood CD4+ T cells and viral load throughout SIV-infection and therapeutic boosting with α-GC+KEX1 Frequencies of peripheral blood CD4+ T cells **(A, B)** and viral load **(C, D)** from Cohort 1 and Cohort 2 comparing vaccinated (VAX) and sham vaccinated (Sham) animals. Data represents the mean ± SD. Statistical significance between VAX and Sham cohorts over time was evaluated by repeated measures mixed modeling analysis using the Geisser-Greenhouse model correction and Sidak correction for multiple comparisons.

iNKT cell-dependent immune activation through therapeutic vaccination with α-GC+KEX1 may in theory increase CD4+ T cell activation and lead to increases in plasma viral load. To test this theory, we monitored peripheral blood CD4+ T cells and plasma viral load throughout SIV-infection. Both cohorts of SIV-infected macaques displayed a typical decline in peripheral blood CD4+ T cells and characteristic chronic-phase plasma viral level. Therapeutic vaccination did not significantly alter CD4+ frequencies (**Figures 2A, B**) or viral load (**Figures 2C, D**) between vaccinated and sham-vaccinated groups in both experimental cohorts. In addition, there was no significant difference in the viral peak, viral setpoint, or chronic CD4+ T cell levels (**Extended Data Tables 1**, **2**). Together, these data indicate that therapeutic boosting with α-GC+KEX1 during the early chronic phase of SIV-infection did not adversely impact circulating CD4+ T cell levels or viremic control in this study.

### Effects of the α-GC+KEX1 on peripheral blood iNKT cell activation and antigen presenting cells

**Figure 3:**
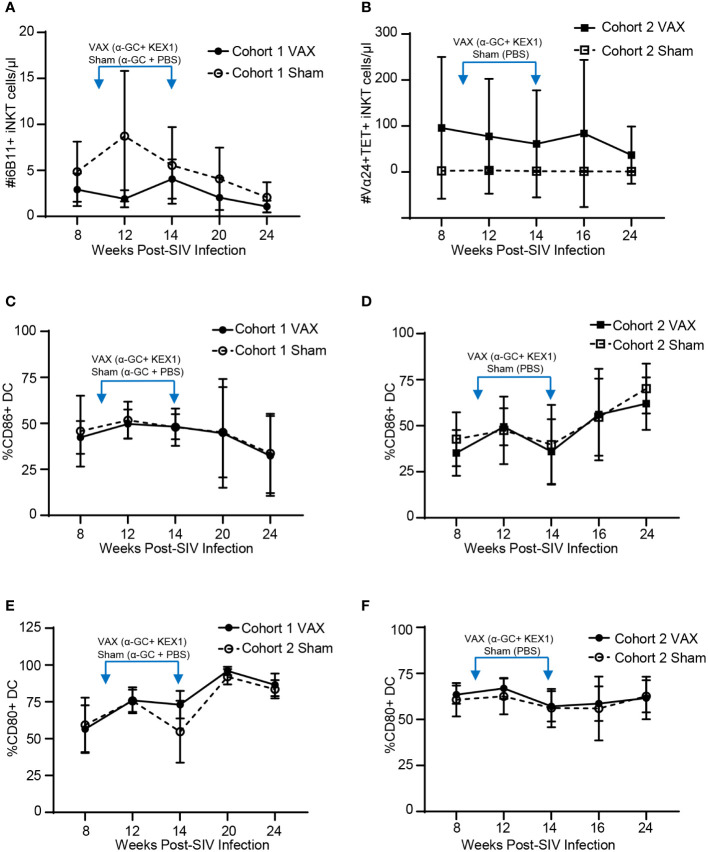
Evaluation of peripheral blood iNKT cells and dendritic cells following therapeutic boosting with α-GC+KEX1. **(A, B)** Cell numbers of peripheral blood iNKT cells, **(C, D)** frequencies of CD86+ dendritic cells (DC) and **(E, F)** frequencies of CD80+ DC in macaques therapeutically boosted at 10 and 14 weeks post-infection (blue arrows) as indicated. iNKT cells in Cohort 1 were identified by staining with the monoclonal antibody against the TCR Vα24-Jα18 (6B11+) where as iNKT cells in Cohort 2 were defined as Vα24+CD1d tetramer PBS-57 (TET+). Data represents the mean ± SD. Statistical significance between VAX and Sham groups over time were evaluated by repeated measures mixed modeling analysis using the Geisser-Greenhouse model correction and Sidak correction for multiple comparisons.

iNKT cells are potent producers of cytokines, which in turn can facilitate the activation of antigen presenting cells such as dendritic cells that promote humoral responses. To understand how therapeutic boosting with α-GC+KEX1 impacts the iNKT cell compartment throughout SIV infection, we monitored iNKT cell numbers in the peripheral blood. iNKT cells in rhesus macaques can express the CD4 co-receptor and are susceptible to CD4-mediated depletion throughout SIV-infection (41). Throughout our studies were unable to detect changes in iNKT cell numbers between vaccinated and sham vaccinated animals in either cohort. Despite the relatively low numbers of iNKT cells present in the peripheral blood of SIV-infected rhesus macaques, iNKT cells remained present throughout course of vaccination and the late chronic phase of infection (**Figures 3A, B**). Importantly, despite the relatively low levels of circulating iNKT cells during SIV infection, humoral immunity was boosted following immunization. When we further evaluated the impact of therapeutic boosting on dendritic cell activation through the expression of the early-stage and late-stage activation markers, CD86 (B7-1) and CD80 (B7-2), we did not observe significant differences in dendritic cell activation between the vaccinated and sham-vaccinated groups within both experiments at the timepoints sampled (**Figures 3C–F**). Collectively, these data indicate that therapeutic boosting with α-GC+KEX1 during the early chronic phase of SIV-infection during the does not significantly deplete iNKT cells or adversely influence dendritic cell activation throughout SIV infection.

### Effects of the α-GC+KEX1 on peripheral CD4+ T effector cell skewing

**Figure 4:**
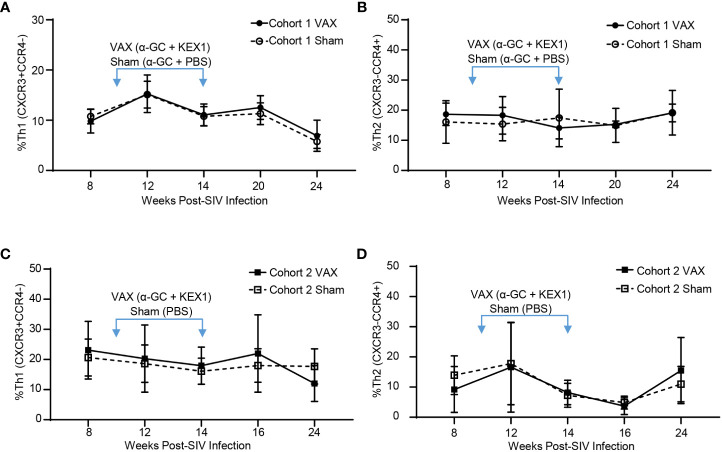
Evaluation of T effector subsets following vaccination with alum+KEX1 and α-GC+KEX1 in Rhesus macaques. Frequency of **(A)** Th1 (CXCR3+CCR4-) and **(B)**, Th2 (CXCR3-CCR4+) effector T cells in Cohort 1 following therapeutic boosting at 10 and 14 weeks post-infection (blue arrows) as indicated. Frequency of **(C)** Th1 and **(D)** Th2 effector T cells in Cohort 2 following therapeutic vaccination. Data represents the mean ± SD. Statistical significance between VAX and Sham cohorts over time was evaluated by repeated measures mixed modeling analysis using the Geisser-Greenhouse model correction and Sidak correction for multiple comparisons.

Achieving a balanced T helper (Th) response following immunization has important implications for immunizing individuals with HIV, as T helper 1 (Th1) responses are critical for viral control (42). We have previously reported that immunization with alum+KEX1 induces a balanced Th1 and T helper 2 (Th2) type response (17). To determine how α-GC+KEX1 promotes humoral immunity during SIV-infection and if this vaccination strategy safely promotes balanced T cell responses, we characterized T helper cell skewing at timepoints surrounding each therapeutic boost throughout SIV-infection. We did not observe significant differences in the frequencies of Th1 or Th2 subsets between vaccinated and sham vaccinated groups in both cohorts at timepoints surrounding therapeutic boosting (**Figures 4A–D**). These data suggest that therapeutic vaccination with α-GC+KEX1 during the early chronic phase of SIV-infection does not negatively impact T helper responses that are essential for viremic control.

### Therapeutic vaccination with α-GC+KEX1 boosts titers in animals with severely reduced CD4+ T cells

**Figure 5:**
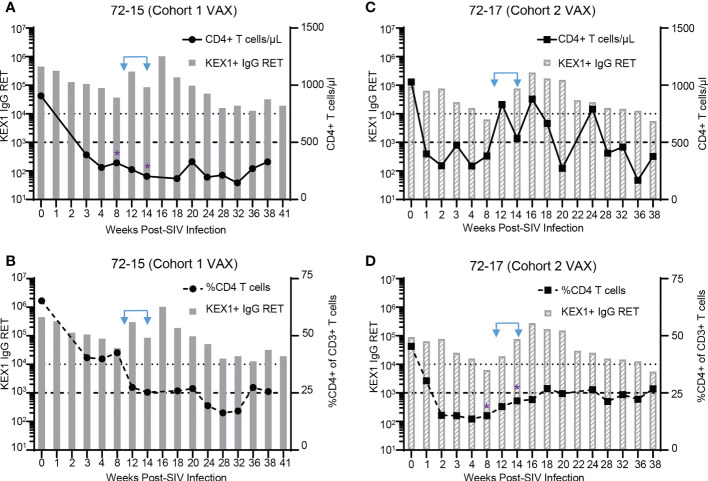
KEX1-antibody responses in therapeutically boosted animals with severe CD4+ T cell depletion. CD4+ T cell depletion profiles and KEX1 antibody responses during SIV in α-GC+KEX1 boosted macaques **(A, B)** 72-15 (Cohort 1 VAX) and **(C, D)** 72-17 (Cohort 2 VAX). **(A, C)** Peripheral blood CD4+ T cell numbers and plasma KEX1 IgG reciprocal endpoint titer (RET) throughout SIV infection. Thick dashed line indicates 500 CD4+ T cells/μL. **(B, D)** Frequencies of peripheral blood CD4+ T cells and plasma KEX1 IgG RET throughout SIV infection. Thick dashed line indicates 25% CD4+ of CD3 T cells. Dotted dashed line indicates the correlate of protection at 10,000 KEX1 IgG RET. Blue arrows indicate therapeutic boosting with α-GC+KEX1 at 10 and 14 weeks post-infections. Purple asterisks indicate timepoints immediately preceding or at the time of therapeutic boosting, where **(A)** CD4+ T cells were less than 500 CD4+ T cells/μL in animal 72-15 (Cohort 1 VAX) or **(D)** the frequency of CD4 T cells was less than 25% in animal 72-17 (Cohort 2 VAX).

Within the vaccinated groups, there were several animals that experienced severe CD4+ T cell depletion at timepoints surrounding vaccination (8 and 14 weeks post-infection), but were still able to elicit robust KEX1-specific IgG titers in response to α-GC+KEX1. These included animals with peripheral blood CD4+ T cells less than 500 cells/µl or had a frequency of CD4+ T cells less than 25% of plasma CD3+ T cells, reflecting conditions of reduced CD4+ T cell help. These animals include 72-15 (**Figures 5A, B**; Cohort 1 VAX) and 72-17 (**Figures 5C, D**; Cohort 2 VAX). At 8 and 14 weeks post-infection, animal 72-15 had 325 and 203 CD4+ T cells/μL, respectively (**Figure 5A**); and 72-17 had a frequency 15% and 22% CD4+ of CD3+ T cells, respectively (**Figure 5D**). Despite diminished CD4+ T cell capacity at 8 and 14 weeks post-infection, α-GC+KEX1 elicited robust titers in both animals. In animal 72-15, KEX1-specific titers increased 27.8-fold between 8 and 16 weeks post-infection (**Figures 5A, C**; **Extended Data Table 1**). In animal 72-17 KEX1-specific titers dropped below the correlate of protection at the 8 week post-infection nadir; however, therapeutic boosting increased titers approximately 43.3-fold between 8 and 16 weeks post-infection (**Figures 5B, D**; **Extended Data Table 2**). These titers were then maintained above the correlate of protection until 36 weeks post-infection. Data from both experiments demonstrate that therapeutic boosting with α-GC+KEX1 during the early chronic phase increases the amplitude and durability of KEX1 IgG titers in profoundly immunosuppressed rhesus macaques.

### A vaccine strategy that boosts pre-existing KEX1-specific memory with α-GC+KEX1 during SIV-infection is protective against *Pneumocystis* co-infection

**Figure 6:**
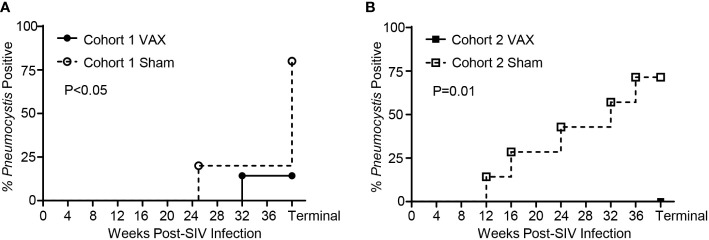
*Pneumocystis* co-infection throughout SIV-infection in vaccinated and sham vaccinated rhesus macaques. **(A)** Incidence of *Pneumocystis* co-infected rhesus macaques in Cohort 1. A diagnosis of *Pneumocystis* co-infection was made through detection of *Pneumocystis* DNA by polymerase chain reaction (first-round PCR) analysis of bronchoalveolar lavage fluid (BAL) or terminal lung homogenate, or by immunohistochemistry staining of FFPE lung sections with anti-*Pneumocystis* antibody 3FC. **(B)** Incidence of *Pneumocystis* co-infection in Cohort 2 was diagnosed through first-round PCR analysis of BAL fluid. Significance was determined by Mantel-Cox analysis.

We previously established that *Pneumocystis* co-infection in rhesus macaques can be reliably diagnosed through the presence of *Pneumocystis* DNA by PCR in bronchoalveolar lavage fluid (BAL) or terminal lung homogenate, or by immunohistochemistry staining with anti-*Pneumocystis* antibody 3F6 (19, 37). Within Cohort 1, at study termination 1 of 7 (14.3%) the vaccinated group developed *Pneumocystis* co-infection compared with 4 of 5 (80%) of sham-vaccinated controls (**Figure 6A**; P<0.05). Similarly, within the Cohort 2, none of the vaccinated animals developed *Pneumocystis* co-infection compared with 5 of 7 (71.4%) sham-vaccinated controls (**Figure 6B**; P=0.01). Of note, the single KEX1-vaccinated animal that developed *Pneumocystis* co-infection, 74-15 (Cohort 1 VAX) experienced a more rapid progression of SIV infection compared to other animals within the same group (CD4+ T cell count <200 cell/µl by 14 weeks post-infection, **Supplemental Figure 1**; chronic CD4+ T cell count of 84 cells/µl, **Extended Data Table 1**) and developed evidence of *Pneumocystis* co-infection by 32 weeks post-infection. Therapeutic vaccination of 74-15 with α-GC+KEX1 successfully induced robust titers at 12 and 16 weeks post-infection that were maintained until 28 weeks post-infection, but notably declined below the correlate of protection at 28 weeks post-infection, a month before a positive *Pneumocystis* diagnosis (**Supplemental Figure 1**). This example further emphasizes the importance of the humoral response in maintaining anti-*Pneumocystis* surveillance. Together, these data indicate that our collective vaccination strategy with therapeutic boosting during the early chronic phase of SIV infection, is protective against *Pneumocystis* co-infection.

## Discussion

HIV infected persons with uncontrolled viremia display diminished CD4+ T cell help and often respond poorly toward conventional vaccination strategies. Several studies indicate that HIV-infected individuals have reduced antibody responses to common recall antigens, including influenza (43), hepatitis A (44), tetanus (45), and pneumococcal (46) vaccines, compared with non-HIV-infected individuals. Therefore, novel immunization strategies in addition to novel vaccine candidates are necessary to protect maintain anti-*Pneumocystis* surveillance and protect highly susceptible HIV-infected populations. Herein, we evaluated the immunogenicity and protective efficacy of a novel anti-*Pneumocystis* therapeutic vaccination strategy in antigen-experienced rhesus macaques during SIV-mediated immunosuppression. We demonstrate that therapeutic boosting with α-GC+KEX1 during the early chronic phase of SIV induced a robust increase in anti-*Pneumocystis* KEX1 IgG titer and an expansion of antigen specific memory B cells. This novel vaccination strategy increased the amplitude and durability of anti-*Pneumocystis* KEX1 titers during SIV infection throughout a ~30 week period following the first therapeutic boost. In a group of antigen-experienced macaques that experienced a precipitous decline in protective titers following SIV infection (Cohort 2-VAX), this strategy restored KEX1 IgG titers in the peripheral blood to levels above the correlate of protection. Moreover, this strategy effectively boosted humoral memory responses even in animals that were profoundly immunosuppressed displaying low peripheral CD4+ T cell numbers or frequencies (<500 CD4+ T cells/µl or <25% CD4+ of CD3+ T cells). Antigen-experienced macaques that were therapeutically boosted during the early chronic phase of SIV-infection were also significantly less likely to develop *Pneumocystis* co-infections (1 of 7 (14.3%) Cohort 1 VAX; 0 of 6 (0%) Cohort 2 VAX) when compared to sham vaccinated controls (4 of 5 (80%) Cohort 1 Sham; 5 of 7 (71.4%) Cohort 2 Sham). In the single KEX1-immunized animal (74-15 Cohort 1 VAX) that developed a *Pneumocystis* co-infection, KEX1-specific titers were initially boosted in response to therapeutic vaccination, but then declined to levels below the correlate protection, likely rendering the animal susceptible. We speculate that if this animal had received an additional therapeutic vaccination with α-GC+KEX1 during the chronic phase of infection, protective titers may have been maintained. These results demonstrate the protective efficacy of our collective vaccination strategy using α-GC+KEX1 therapeutic boosting and further emphasizes the importance of KEX1-specific antibodies in maintaining anti-*Pneumocystis* surveillance. These data support the concept that protective, protein-vaccine-induced B-cell memory responses can be recalled during SIV-mediated immunosuppression through therapeutic boosting with α-GC.

In addition to evaluating the effect of therapeutic vaccination with α-GC+KEX1 on humoral immunity, we examined it’s impact on the peripheral CD4+ T cells, T helper skewing, and viral control. During HIV infection, Th1 responses play a critical in viral control (42). Following SIV infection, all of the macaques included in our analysis displayed a characteristic decline in the frequency and cell number of peripheral blood CD4+ T cells. Therapeutic vaccination effectively boosted plasma IgG titers without negatively impacting peripheral blood CD4+ T cell frequency, T helper skewing, or plasma viral load. These results represent an optimal therapeutic balance whereby anti-*Pneumocystis* humoral immunity is safely boosted without negatively impacting viremic control.

As an extension of our investigation into the impact of α-GC+KEX1 vaccination on humoral immunity during SIV-immunosuppression, we also examined peripheral blood iNKT cell numbers and their impact on DC activation. iNKT cells are potent producers of cytokines, which in turn can facilitate the activation of antigen presenting cells that promote humoral responses. Healthy rhesus macaques have relatively low number of iNKT cells in the peripheral blood compared to human populations (41), and subsets of iNKT cells that express the CD4 co-receptor are susceptible to SIV-mediated depletion (41). As an innate activator, α-GC typically induces peak iNKT cell activation within 8-12hrs of administration, peak clonal expansion within ~72hrs, followed by contraction to homeostatic levels within ~1-2 weeks (23). Many of our sample timepoints were beyond the peak window of innate activation and proliferation. This could explain why we were unable to observe transient differences in either iNKT cell numbers or dendritic cell in response to therapeutic vaccination at the timepoints analyzed. We were also unable to observe significant differences in iNKT or CD4+ T cell activation through the expression of CD107a or CD38 and HLADR, respectively following α-GC+KEX1 boosting (data not shown). The longitudinal changes we did observe were most likely due to background inflammation caused by SIV-infection. Although we were unable to directly demonstrate that α-GC+KEX1 induces humoral boosting through iNKT cell activation, both iNKT cell populations and DC were not negatively impacted by therapeutic boosting further supporting the safety of this approach.

Several studies have demonstrated enhanced anti-pathogen and anti-tumor immunity following immunization with α-GC (26, 27, 47). iNKT cells can provide B cell help through cognate interaction with B cells, or non-cognate help by promoting priming of effector and memory T cells by iNKT cell-licensed DC (48). Skewing toward cognate B cell help has been typically observed in vaccination strategies where antigens are directly conjugated to α-GC or carried by the same nanoparticle with α-GC (28, 49), or in the absence of CD4+ T cell help (50). In contrast, non-cognate help has been observed following vaccination with α-GC mixed with unconjugated protein antigens (47). Whether cognate iNKT cell help promotes the establishment of long-term memory humoral memory responses remains controversial. Some groups have reported that cognate help strategies using pneumococcal capsular polysaccharides in naïve animals induce prolonged antibody responses, class switching, affinity maturation, and long-lasting B cell memory (27, 51). Whereas others have reported that cognate iNKT cell help generates limited humoral memory responses following vaccination of naïve animals (29, 50) and induces the expansion of IL-10-regulatory B cells that may limit the formation of long-term memory (29). In the scope of the current study, it is likely that both cognate and non-cognate iNKT cell help occur following therapeutic vaccination with α-GC+ KEX1. This may be due to the use of a vaccine strategy in which α-GC is mixed with unconjugated KEX1 peptide, and administration of α-GC+KEX1 under conditions of reduced CD4+ T cell help due to SIV mediated depletion. Previous studies investigating the impact of iNKT cell cognate-help on humoral memory were performed through vaccination of naïve animals. In contrast, animals therapeutically boosted with α-GC+KEX1 during SIV infection within this study, were antigen-experienced through a combination of vaccination with alum+KEX1 prior to SIV-infection and natural exposure, as determined by baseline anti-KEX1 IgG titers. Exposure to *Pneumocystis* is common and most individuals have positive serology for *Pneumocystis* antigens by early childhood (12–14). Most human adults (both HIV positive and HIV negative) are KEX1-seropositive as a result of regular exposure to this ubiquitous pathogen (1, 20). The current study supports the concept that vaccination with α-GC+KEX1 is capable of inducing expansion of KEX1-specific IgG memory B cells and maintenance of elevated plasma KEX1 IgG titers in the context of SIV-induced immune dysregulation and diminished CD4+ T cells numbers.

This study builds upon previous work in several ways. To our knowledge, this is the first study to demonstrate that vaccination with α-GC can be used to boost titers against a recall antigen during SIV-immunosuppression. In addition, these data demonstrate that therapeutic vaccination with α-GC and a protein antigen can be used to safely and effectively boost protective titers against a respiratory pathogen in a severely immunocompromised host with uncontrolled SIV-viremia. Our collective vaccination strategy helped to maintain protective surveillance against *Pneumocystis* co-infection for more than 6 months in SIV-infected macaques. The findings from this study are clinically relevant because they may be used to develop a novel vaccination strategy to protect populations at increased risk of *Pneumocystis* infection during the early chronic phase of HIV infection, particularly among those who have not achieved viremic control through cART. The efficacy of therapeutic boosting with α-GC+KEX1 alone in the context of SIV or HIV immunosuppression is still unknown. Moreover, therapeutic vaccination with α-GC+KEX1 may be improved through supplementation with conventional adjuvant strategies. Studies to evaluate the potential use of α-GC+KEX1 alone or and in combination with conventional adjuvants in immunosuppressed macaques are ongoing. Observations from the current and continued studies can potentially be applied to both HIV and non-HIV infected persons at risk of developing *Pneumocystis* and toward other opportunistic pathogens in immunosuppressed individuals.

## Data availability statement

The raw data supporting the conclusions of this article will be made available by the authors, without undue reservation.

## Ethics statement

The animal study was reviewed and approved by University of Georgia-Institutional Animal Care and Use Committee (IACUC).

## Author contributions

WR, FS, HK, and KN conceived and planned the experiments. WR, FS, LB, and ER performed the experiments and data analysis. KN supervised the project and assisted in data interpretation. WR and KN wrote the manuscript in consultation with FS, HK, LB, and ER. All authors contributed to the article and approved the submitted version.
