# Supplementary material for: A therapeutic vaccine strategy to prevent Pneumocystis pneumonia in an immunocompromised host in a non-human primate model of HIV and Pneumocystis co-infection
Source: Front Immunol. 2022 Dec 6;13:1036658. doi: 10.3389/fimmu.2022.1036658 (PMC9763597; doi:10.3389/fimmu.2022.1036658)
Supplement: Supplementary file 3 [file Presentation_1.pptx]

## Slide 1
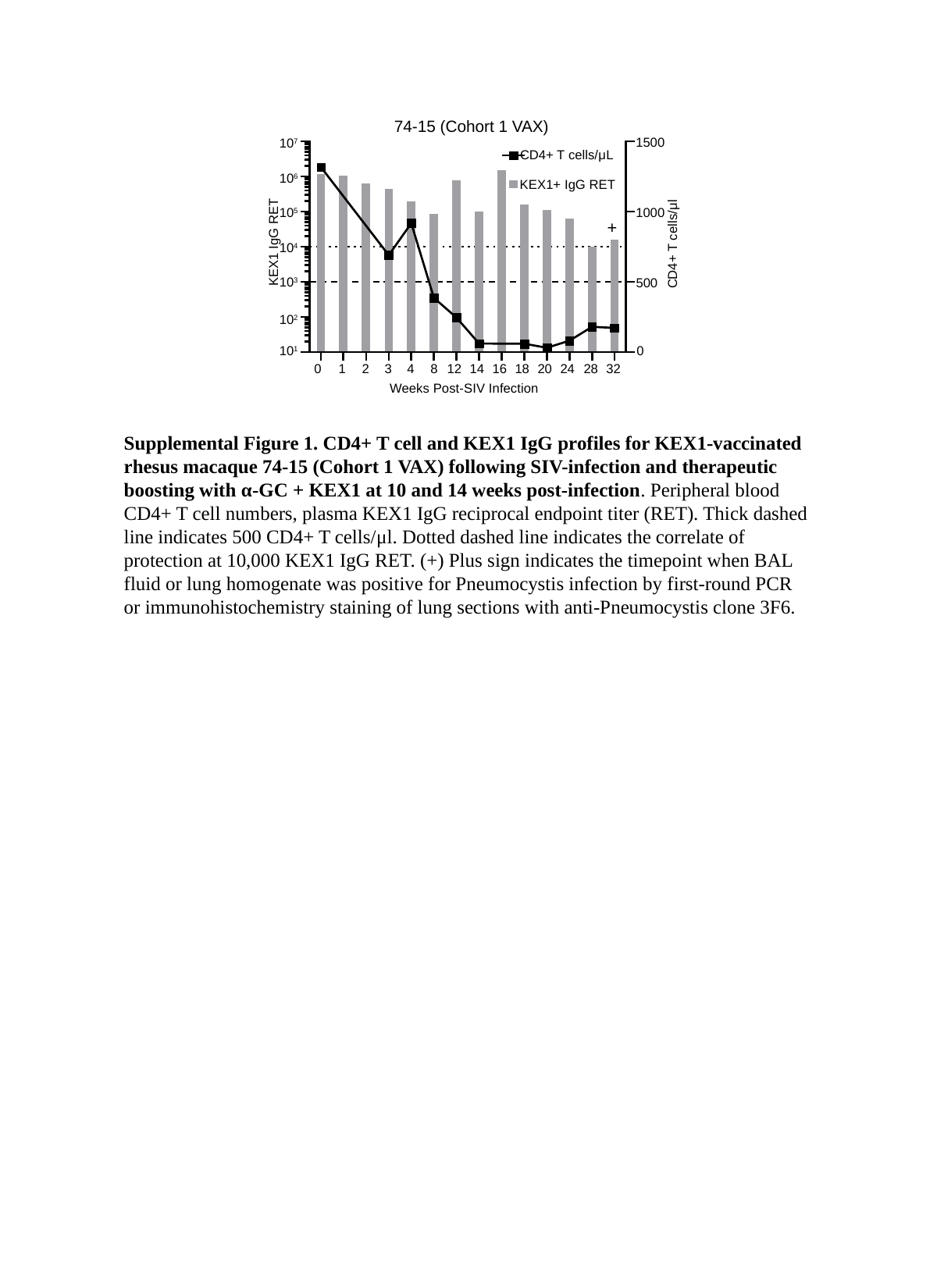

74-15 (Cohort 1 VAX)
1500
107
CD4+ T cells/μL
106
KEX1+ IgG RET
105
1000
+
KEX1 IgG RET
CD4+ T cells/μl
104
103
500
102
101
0
0
1
2
3
4
8
12
14
16
18
20
24
28
32
Weeks Post-SIV Infection
Supplemental Figure 1. CD4+ T cell and KEX1 IgG profiles for KEX1-vaccinated rhesus macaque 74-15 (Cohort 1 VAX) following SIV-infection and therapeutic boosting with α-GC + KEX1 at 10 and 14 weeks post-infection. Peripheral blood CD4+ T cell numbers, plasma KEX1 IgG reciprocal endpoint titer (RET). Thick dashed line indicates 500 CD4+ T cells/μl. Dotted dashed line indicates the correlate of protection at 10,000 KEX1 IgG RET. (+) Plus sign indicates the timepoint when BAL fluid or lung homogenate was positive for Pneumocystis infection by first-round PCR or immunohistochemistry staining of lung sections with anti-Pneumocystis clone 3F6.
